# Sexual Dimorphism in Balance and Coordination in p75NTR^exonIII^ Knock-Out Mice

**DOI:** 10.3389/fnbeh.2022.842552

**Published:** 2022-02-24

**Authors:** Mahdi Abbasian, Annick Langlois, Julien Gibon

**Affiliations:** Biology Department, University of British Columbia Okanagan, Kelowna, BC, Canada

**Keywords:** p75NTR, mice, sex, behavior, balance, coordination

## Abstract

The p75 neurotrophin receptor (p75NTR) is implicated in various biological functions during development and adulthood. Several animal models have been developed to identify the roles of p75NTR *in vivo* and *in vitro*. P75NTR^ExonIII^ knock-out mice are widely used to study the neurotrophin receptor and its signaling pathways. Similar to other models of p75NTR knock-out (p75NTR^Exon IV^ KO) or conditional knock-out (p75NTR^fl/fl^) mice, p75NTR^ExonIII^ knock-out mice present severe abnormalities in walking, gait, balance and strength. The present study identifies a sexual dimorphism in the p75NTR^ExonIII^ knock-out strain regarding balance and coordination. Using Kondziela’s inverted grid test, we observed that p75NTR^ExonIII^ knock-out males performed poorly at the task, whereas p75NTR^ExonIII^ knock-out females did not exhibit any defects. We also observed that female p75NTR^ExonIII^ knock-out mice performed significantly better than male p75NTR^ExonIII^ knock-out mice at the beam balance test. There were no differences in strength, skin innervation, or the number of ulcers on the toes between p75NTR^ExonIII^ knock-out males and females. The literature regarding the role of p75NTR in behavior is controversial; our results suggest that studies investigating the role of p75NTR *in vivo* using p75NTR knock-out mice should systematically report data from males and females.

## Introduction

The p75 Neurotrophin Receptor (p75NTR), also known as CD271, belongs to the tumor necrosis factor receptor superfamily (TNFRSF16). p75NTR binds to all neurotrophins and proneurotrophins (Roux and Barker, 2002; Gibon and Barker, 2017), as well as amyloid-beta peptides (Yaar et al., 1997) and is implicated in various processes, from cell survival to cell death (Roux and Barker, 2002). p75NTR is also involved in synaptic refinement in the central nervous system, where it plays a critical role in the long-term depression of synapses in the hippocampus (Woo et al., 2005), and in the regulation of neuronal activity (Gibon et al., 2015, 2016) among many other functions (Chao, 2003). These discoveries have been largely driven by the development of animal models that lack the full-length p75NTR expression. The p75NTR^ExonIII^ knock-out (p75NTR^ExonIII^ KO) model made by Lee et al. (1992) is the most commonly used in the field. These animals lack the receptor’s extracellular domain but express a spliced variant (s-p75NTR) with the intracellular domain (ICD) (Paul et al., 2004). Another model, p75NTR^ExonIV^ knock-out (p75NTR^exonIV^ KO), was later produced by von Schack et al. (2001). Although initially described as a complete knock-out, it was subsequently shown that p75NTR^ExonIV^ KO mice also express a truncated p75NTR containing the transmembrane domain and the ICD (Paul et al., 2004). Even though the two models are not complete knock-out, they have similarities. For example, both p75NTR^ExonIII^ KO and p75NTR^exonIV^ KO mice have a “mud-walking” gait (Lee et al., 1992; von Schack et al., 2001). The exact reason for these walking defects is unclear, but several exciting findings have been made. Specifically, p75NTR KO mice have a dramatic reduction in dorsal root sensory neurons, which correlates with a substantial decrease in sensory innervation of the paws (Murray et al., 1999; Gjerstad et al., 2002). Interestingly, the walking defect has also been observed in a model where p75NTR was conditionally deleted, using the p75NTR^fl/fl^ model produced by Bogenmann et al. (2011). For instance, deletion of p75NTR only in the granule cell layer or the cerebellum recapitulates the gait defect (Zanin et al., 2016). Recent findings suggest that p75NTR is also critical for forming the neuromuscular junction and normal strength (Reddypalli et al., 2005; Dokter et al., 2015; Pérez et al., 2019). In summary, many research groups have shown that p75NTR KO animals have dysfunctions in gait, strength, balance, and various neuronal processes. However, the studies mentioned above have only tested the dysfunctions in male p75NTR KO (Dokter et al., 2015) or do not mention the sex of the animals used in the studies (Lee et al., 1992; Murray et al., 1999; Peterson et al., 1999; Gjerstad et al., 2002; Reddypalli et al., 2005; Zanin et al., 2016; Pérez et al., 2019). A recent finding by Puschban et al. (2016) using p75NTR^ExonIV^ KO mice, suggests a sexual dimorphism exists regarding the hyperactivity and the level of anxiety in these mice. Interestingly, we observed that male and female p75NTR^ExonIII^ KO mice behave very differently in their home cages, with females often climbing their cage lid while males stay on their bedding. This observation led us to investigate if a sexual dimorphism exists in the p75NTR^ExonIII^ KO mice regarding their balance, gait and strength. In the present study, we discovered that p75NTR KO females outperformed p75NTR KO males in the inverted grid test and balance beam test. We found no differences in strength between p75NTR KO females and males, and both sexes showed a very significant defect in skin innervation, presenting with small, characteristic ulcers on their toes. Our results suggest that future studies investigating the role of p75NTR in behavior should consider reporting data from both males and females to improve reproducibility between laboratories.

## Materials and Methods

### Animals

p75NTR Exon III KO mice described by Lee et al. (1992) were maintained in a C57BL/6NCrl background (Charles River, Montreal, Canada). Breeding was performed by crossing heterozygote animals. Wild-type and p75NTR KO littermates were used for the study, and genotyping was confirmed using standard PCR. Animals were maintained in a 12 h light/dark cycle and had access to food, water, *ad libitum*. Efforts were made to reduce animal handling and use. All animal procedures and experiments were approved by the UBC Animal Care Committee.

### Inverted Grid Test

The Kondziela’s inverted screen test was performed with the maximum criterion of 60 s, with some modification from Deacon (2013). A mouse was placed in the center of the wire mesh, and the grid was inverted. The time spent in contact with the grid was recorded. Each animal was tested on three separate trials, with 10 min between each trial. The average time spent in contact with the grid is reported.

### Weights Test

Seven weights were used to perform the test. The weights used were: 16, 28, 39, 52, 65, 78, 92 g. Only weights held for at least 3 s with the forelimbs were considered successful (Deacon, 2013). The test was stopped after 3 s. The following scores were assigned: 1 = successfully holds 16 g, 2 = successfully holds 28 g, 3 = successfully holds 39 g, 4 = successfully holds 52 g, 5 = successfully holds 65 g, 6 = successfully holds 78 g, and 7 = successfully holds 92 g.

### Beam Walking Test

Beams were 1 m long and positioned 1 m above the ground, with a safe zone at one end where the cage was located, containing home cage bedding and food. Three different beam diameters were used: 3, 2.5, and 1.5 cm. Each mouse was trained to perform the task on at least three consecutive trials with the 3 cm beam, starting 10 cm from the safe location, then 50 cm, and finally at the start of the beam. Mice were not tested on the smaller beams before crossing the 3 cm diameter beam entirely. During the test, mice were first positioned at the end of the 3 cm diameter beam, and the number of foot slips was recorded. Mice were then placed in their home cage before being tested on the 2.5 cm diameter beam and the 1.5 cm diameter beam. The number of foot slips per beam was reported for each genotype and sex.

### Immunohistochemistry

OCT-embedded hind-paw skin was sectioned transversely (30 μm), and every third section was collected onto separate gelatin-coated slides. Sections were air-dried overnight. The sample was rehydrated in PBS (Wisent, Canada), permeabilized (0.3% TritonX-100, 5% Normal Goat Serum in PBS), blocked and immunostained with rabbit anti-Calcitonin Gene-Related Peptide (Sigma C8198, Canada) diluted at 1:2500 in blocking solution (0.3% TritonX-100, 5% Normal Goat Serum in PBS) and incubated overnight at 4°C. Slides were then washed with PBS and incubated with goat anti-rabbit conjugated to Alexa Fluor 488 (Jackson Immunoresearch Laboratories, United States) 3 h at room temperature in the dark (secondary antibodies were diluted at 1:1000 in blocking solution). Sections were counterstained with 1 μg/ml of DAPI and mounted with Fluoroshield mounting media (Millipore-Sigma, F6182, Canada). Skin sections were imaged using a Leica DMi8 confocal microscope and LAS X software at 40×. Immunolabeled intraepidermal CGRP-positive fibers were manually and blindly counted. Three sections per animal were analyzed, with three pictures per slice.

### Statistical Analysis

In each experiment, two variables were considered: sex (male or female) and genotypes (wild-type or p75NTR knock-out). A two-way ANOVA followed by Tukey multiple comparison test was performed to test the significance of the data, with *p* < 0.05 considered as significant.

## Results

**FIGURE 1:**
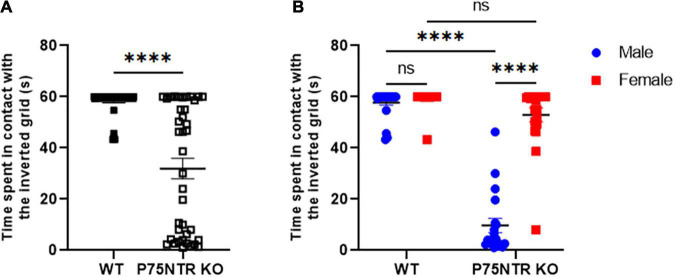
Female p75NTR KO performed better than male p75NTR KO at the inverted grid test. **(A)** Each mouse was tested three times on the inverted grid test. Each point represents the average time a mouse spent in contact with the grid. The maximum time allowed on the grid was 60 s (*n* = 43 WT and *n* = 39 p75NTR KO, ****p < 0.0001 *T*-test). **(B)** Same data as in panel **(A)** but separated by sex. Blue dots represent males; *n* = 24 mice WT and *n* = 19 p75NTR KO. Red squares represent females; *n* = 19 for WT and *n* = 20 for p75NTR KO. The black bar represents the mean for each group. ns, non-significant. ****p < 0.0001 two-way ANOVA followed by Tukey multiple comparison test.

P75NTR^ExonIII^ knock-out (p75NTR^ExonIII^ KO) mice have been widely used to study the role of p75NTR in multiple contexts. The first description by Lee et al. (1992) clearly shows that these mice have issues with the morphology of their paws. Swollen or missing digits and missing nails are very common in p75NTR KO. Additionally, several groups have reported that these mice present a defect in gait, sometimes described as “mud-walking gait”(Puschban et al., 2016). However, there is no consensus within the field about how the defect in mice’s gait affects their ability to perform tasks requiring strength, balance and coordination (Lee et al., 1992; Peterson et al., 1999; Reddypalli et al., 2005; Pérez et al., 2019). We decided to test p75NTR^ExonIII^ KO animals on a Kondziela’s inverted grid test to tackle this question. During the task, the grid is inverted, and the animal must hold its body weight while the head is facing down (inverted), requiring the animal to coordinate its movements and actions simultaneously. Wild-type animals succeed very easily at the task (Figure 1A); the maximum time before removing the animal from the grid was 60 s in our experiment. However, when we tested p75NTR KO mice, they showed a range of time spent in contact with the grid from 0 to 60 s. It was discovered that while some animals could not perform the task, others succeeded with no apparent issues. Unexpectedly, we realized that the variation in the results was due to the sex of the animals: male**s** p75NTR KO were not able to perform the task, while female**s** p75NTR KO showed almost no defect in the Kondziela’s test (Figure 1B).

**FIGURE 2:**
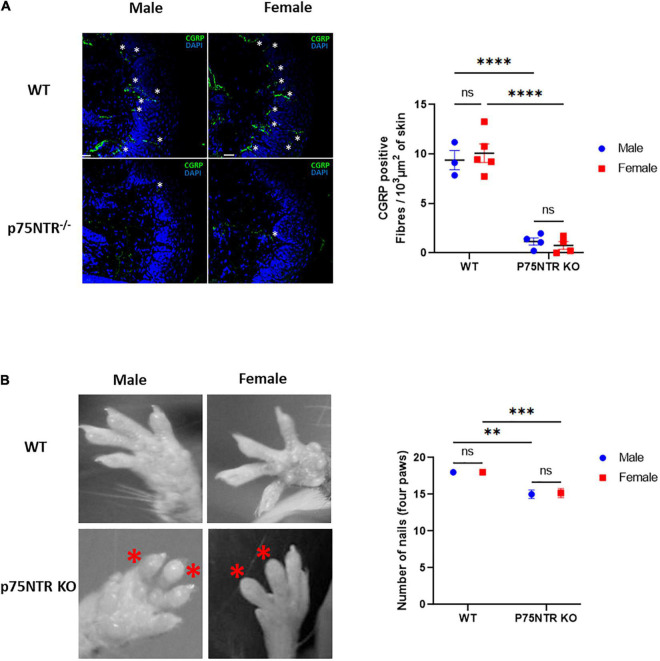
Female and male p75NTR KO show similar defects in skin innervation and toes morphology. **(A)** The number of CGRP positive fibers (indicated with white*) crossing the epidermis was quantified using immunohistochemistry and confocal imaging. Three sections, from 3 WT males, 5 WT females, 4 p75NTR KO males, and 4 p75NTR females were quantified. Each dot represents the average number of fibers crossing the epidermis (expressed in μm^2^). Horizontal bars represent the mean for each genotype. ns, non-significant. ****p < 0.0001 two-way ANOVA followed by Tukey Multiple comparison test. **(B)** The four paws of WT and p75NTR KO males and females were screened for toe morphology. Male and female P75NTR KO mice show a similar swelling pattern with several missing nails. Total number of nails were quantified and compared with a two-way ANOVA followed by Tukey Multiple comparison test,***p < 0.001,**p < 0.01 (*n* = 7 WT males, 4 WT females, 3 p75NTR KO males, and 6 p75NTR KO females).

The p75NTR^ExonIII^ knock-out has a defect in sensory skin innervation due to a reduced number of dorsal root ganglion neurons (Murray et al., 1999; Gjerstad et al., 2002). However, we could not find if that effect is sex-dependent in the literature. To investigate this, we quantified the innervation of the skin of wild-type and p75NTR KO male and female mice using immunohistochemistry. Calcitonin Gene-Related Peptide was used as a sensory terminal marker. Figure 2A shows that male and female p75NTR KO have a substantial defect in skin innervation. We then looked at the gross morphology of the toes, and could not detect differences between male and female p75NTR KO (Figure 2B). Both sexes showed swelling and missing nails, as previously described (Lee et al., 1992). These morphological observations could not explain the sexual dimorphism observed in the inverted grid test.

**FIGURE 3:**
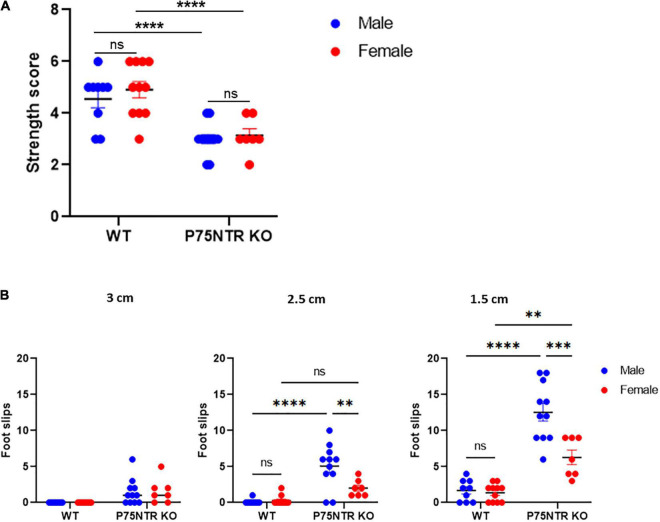
Female and male p75NTR KO have similar strength defects, but females p75NTR KO showed better balance and coordination than males p75NTR KO on the beam balance test. **(A)** The strength of WT and p75NTR KO mice was tested using a weight test *n*: 9 WT male, 11 WT female, 12 p75NTR KO male, and 7 p75NTR female. ns; non-significant ****p < 0.0001 two-way ANOVA followed by Tukey Multiple comparison test. **(B)** A beam walking test using three different diameters (3, 2.5, and 1 cm) was used to test the balance and coordination of 9 WT males, 11 WT females, 11 p75NTR KO males, and 7 p75NTR KO females. The number of foot slips was scored. ns, non-significant. **p < 0.01, ***p < 0.001, ****p < 0.0001 two-way ANOVA followed by Tukey Multiple comparison test.

The inverted grid test is often used to test the strength of mice (Deacon, 2013). However, while the grid rotates, the task also requires perfect balance and coordination. We first tested whether males and females p75NTR KO show differences in forepaws strength using a simple weight test. As previously described, we observed that p75NTR KO mice have an overall defect in strength (Pérez et al., 2019), but we could not detect a difference based on the sex of the animal (Figure 3A). We reasoned that if the difference observed in the Kondziela’s test is not due to strength, it may be due to differences in balance and coordination. A beam walking task was used to test this hypothesis. Mice were trained on a 3 cm diameter beam; WT and p75NTR^ExonIII^ KO performed very well. However, when we reduced the size of the beam diameter to 2.5 cm, we observed that p75NTR^ExonIII^ KO showed more foot slips than WT animals. This result confirmed the gait defect of the p75NTR^ExonIII^ KO, but we also observed a significant difference between male and female p75NTR^ExonIII^ KO mice. As with the inverted grid test, females p75NTR^ExonIII^ KO performed better than males p75NTR^ExonIII^ KO. The difference was still evident when we used a smaller beam (1.5 cm diameter), where females p75NTR^ExonIII^ KO outperformed males p75NTR^ExonIII^ KO (Figure 3B). However, both male and female p75NTR^ExonIII^ KO mice performed poorly compared to WT, indicating that p75NTR^ExonIII^ KO mice have a strong balance and coordination defect.

## Discussion

p75NTR^ExonIII^ KO mice are used by many laboratories globally. As a result, this strain has been instrumental in discovering a variety of roles associated with p75NTR. Specifically, p75NTR is involved in synaptic functions such as long-term depression of synapses (Gibon and Barker, 2017), in Alzheimer’s disease (Mufson et al., 2019; Patnaik et al., 2020), in metabolism (Baeza-Raja et al., 2016) and many other key biological processes (Roux and Barker, 2002). p75NTR is a complex receptor that acts with several co-receptors and binds to all forms of neurotrophins and proneurotrophins (Gibon and Barker, 2017) in a cell-dependent manner. Most of the literature regarding the study of p75NTR in mice has not reported the sex of the animal used in experiments. However, it was recently observed by Puschban et al. (2016) that a sex difference exists in the p75NTR^ExonIV^ KO model with respect to their activity and performance at several behavioral tests. Female p75NTR^ExonIV^ KO mice were more active than WT females in the open-field test and were less anxious. Males p75NTR^ExonIV^ KO performed similarly to wild-type males. In this study, Puschban et al. (2016) addressed the difference between KO and WT animals, but not the differences within the KO animal groups, leaving open the question of whether p75NTR KO males and females perform statistically differently on these tasks. The p75NTR^ExonIV^ KO expresses a truncated version of p75NTR, containing a small fragment of the extracellular domain, the transmembrane domain and the ICD (Paul et al., 2004). It was shown that the p75NTR^ExonIV^ products found in the knock-out animal could cause apoptosis in PC12 cells (Paul et al., 2004). Our study’s data suggest that future research should address if a similar sexual dimorphism in balance and coordination is observed in p75NTR^ExonIV^ KO mice.

The p75NTR^ExonIII^ KO mice express a spliced form of p75NTR (s-p75NTR); however, its detection *in vivo* is controversial, and it has not been demonstrated that this fragment is active *in vivo*. While these two models are not entirely knock-out animals, similar observations have been made regarding their gait. Both strains show defects in gait, called “mud-walking gait” (von Schack et al., 2001). The first description of the p75NTR^ExonIII^ KO revealed that these animals have defects in sensory innervation and develop ulcers at the tip of their toes (Lee et al., 1992). While not directly measured, Lee et al. (1992) noticed that p75NTR^ExonIII^ KO have some impairment in their ability to grasp cage lids.

In the present study, we questioned whether the p75NTR^ExonIII^ KO could be used to study ataxia. Based on the discrepancy in the literature, we decided to test our colony of p75NTR^ExonIII^ KO (maintained in a C57BL6 background) with a simple inverted-grid test. Surprisingly, the variability in our data was extreme, from animals not able to perform the task and falling as soon as the grid was inverted to animals showing no defect at all. We observed a clear pattern only when the data points were separated by sex, with males incapable of doing the task while females performed similarly as wild-type animals. The Kondziela’s inverted grid test measures an animal’s strength and coordination. Additionally, we used a weight test to measure the strength of the animals and confirmed the previous observation that p75NTR^ExonIII^ KO animals are weaker than WT animals (Reddypalli et al., 2005; Pérez et al., 2019). We could not detect differences between males and females p75NTR^ExonIII^ KO. The lack of differences in strength prompts us to look at the sensory nervous system of p75NTR^ExonIII^ KO to explain the results of the Kondziela’s test. Several groups have shown that p75NTR^ExonIII^ KO mice have a severe defect in skin innervation and a reduced number of dorsal root ganglion neurons (Murray et al., 1999; Gjerstad et al., 2002). It has also been well described that p75NTR^ExonIII^ KO mice have ulcers on their toes (Lee et al., 1992). We confirmed these observations in our colony and did not observe differences between males and females p75NTR^ExonIII^ KO. Males and females showed almost no CGRP positive fibers in the epidermis, and several toes presented swelling or small injuries. p75NTR knock-out mice have been described to have a reduced mechanical pain sensitivity (Lee et al., 1992; Bergmann et al., 1997) compared to wild-type animals. However, it is unknown if a difference exists between male and female p75NTR KO mice. Interestingly recent research demonstrated that male and female mice perceived pain differently (Sorge et al., 2011). Future research should address if male and female p75NTR knock-out mice have an identical pain threshold.

Using a beam walking task, we tested the hypothesis that the inverted grid test reveals balance and coordination differences between male and female p75NTR^ExonIII^ KO. Wild-type animals performed very well, whereas p75NTR KO animals showed significantly more foot slips on every beam. Similarly, as demonstrated in the inverted grid test, we observed that females p75NTR KO outperformed males in the beam walking test.

The vestibular system and cerebellum are crucial to maintaining optimal balance and coordination. The vestibular system connects with the vestibular ganglion, which sends primary afferents directly to the cerebellum and secondary afferents to the vestibular nuclei. Primary and secondary afferents synapse with unipolar brush cells within the cerebellum, which connect with granule cells that ultimately contact Purkinje cells (Ango and Dos Reis, 2019). Interestingly, Zanin et al. (2016) showed that the conditional deletion of p75NTR only in cells from the external granule layer recapitulate the balance defect observed in the p75NTR^ExonIII^ KO. These results demonstrate the critical role of p75NTR in the development of the cerebellum. Although the role of p75NTR in the inner ear is unknown, p75NTR is expressed in glial cells (mainly Schwann cells) within the Cochlea (Liu et al., 2012). Despite our study not providing a mechanism to explain why females p75NTR^ExonIII^ KO have a better balance and coordination than males p75NTR^ExonIII^ KO, the tests performed combined with the literature suggest that the mechanism involves the neuronal system controlling proper balance and coordination. We observed a similar reduction in the number of nerve fibers in the paws of p75NTR KO male and female mice; however, our work does not exclude the possibility that the signaling pathways required for proprioception may be differently affected in males and females p75NTR KO.

In our experiments, we did not find any morphological differences between male and female wild-type mice. These results would have justified the use of only one of the two sexes in the investigation -most often, males to keep the females for breeding purposes- however, our new data strongly emphasize the importance of testing male and female mice and reporting data by sex and not only by genotype. The literature regarding the effect of p75NTR deletion on behavior is controversial. For instance, several groups have studied the impact of p75NTR deletion on memory using the p75NTR^ExonIII^ mice. In these studies, the sex of the animals is not reported (Barrett et al., 2010), or only one of the sexes is used (Peterson et al., 1999; Greferath et al., 2000; Wright et al., 2004; Catts et al., 2008; Martinowich et al., 2012; Dokter et al., 2015; Gibon et al., 2015). These studies’ conclusions are controversial, with groups showing that p75NTR KO mice outperformed wild-type animals in spatial memory test or short-term memory (Greferath et al., 2000; Barrett et al., 2010; Gibon et al., 2015), while other groups found opposite results (Peterson et al., 1999; Wright et al., 2004; Dokter et al., 2015) or very little to no effect (Catts et al., 2008). Interestingly, the study from Greferath et al. (2000) only used females and found that p75NTR KO mice outperformed wild-type females in spatial memory. The studies from Peterson et al. (1999), Wright et al. (2004), and Dokter et al. (2015) used only males and found that p75NTR KO mice did not learn the spatial memory task and performed very poorly compared to wild-type male mice. Different colonies and potentially different genetic backgrounds are present in the strains used by these groups, but in light of our new data, it is interesting to note that female and male p75NTR KO may perform very differently in other tasks than balance and coordination.

To conclude, our study suggests that groups studying the impact of p75NTR deletion on behavior should systematically test male and female knock-out animals to improve the reproducibility of the data between laboratories.

## Data Availability Statement

The original contributions presented in the study are included in the article/supplementary material, further inquiries can be directed to the corresponding author.

## Ethics Statement

The animal study was reviewed and approved by the UBC Animal Care Committee.

## Author Contributions

MA performed experiments, analyzed data, and reviewed the manuscript. AL performed experiments and reviewed the manuscript. JG performed experiments, analyzed data, and wrote the manuscript. All authors contributed to the article and approved the submitted version.

## Conflict of Interest

The authors declare that the research was conducted in the absence of any commercial or financial relationships that could be construed as a potential conflict of interest.

## Publisher’s Note

All claims expressed in this article are solely those of the authors and do not necessarily represent those of their affiliated organizations, or those of the publisher, the editors and the reviewers. Any product that may be evaluated in this article, or claim that may be made by its manufacturer, is not guaranteed or endorsed by the publisher.
